# Vasospasm-Induced Spreading Depolarization and/or Spreading-Depolarization-Induced Vasospasm After Subarachnoid Hemorrhage

**DOI:** 10.1007/s12028-021-01373-3

**Published:** 2021-10-26

**Authors:** Jens P. Dreier

**Affiliations:** 1 Center for Stroke Research Berlin and Departments of Experimental Neurology and Neurology, Charité–Universitätsmedizin Berlin, Corporate Member of Freie Universität Berlin, Humboldt-Universität Zu Berlin, and Berlin Institute of Health, Berlin, Germany; 2 Bernstein Center for Computational Neuroscience Berlin, Berlin, Germany; 3 Einstein Center for Neurosciences Berlin, Berlin, Germany

The discovery that spreading depolarizations (SDs) can be acutely induced by injection of autologous blood into the subarachnoid space or by micropuncture of a cortical vessel dates back to Hubschmann and Kornhauser [1], who demonstrated this in cats in 1980. In the last sentence of the abstract, the two authors explicitly hypothesized that cortical cells rather than blood vessels are the primary targets in the initial stages of subarachnoid hemorrhage (SAH). Furthermore, in the introduction they wrote, “Objections to the concept linking the vasospasm directly with the neurological deficit have been raised on theoretical grounds, and the clinical relationship between the presence of vasospasm and the neurological deficit in patients has been poor.” These statements alone show that the controversy about the pathogenesis of early and delayed focal brain damage after SAH has a long and checkered history.

As for SD, we now know, on the basis of a broad experimental evidence, that SD is probably the most important pathway leading to neuronal cytotoxic edema and diffusion restriction in the gray matter of the central nervous system [2]. Increasingly, clinicians and neuroscientists also understand that SD can vary widely in duration, that neuronal survival probability decreases with increasing duration of SD, and that SD may be associated not only with one stereotypic change in spontaneous brain activity but also with various changes, such as nonspreading activity depression, spreading activity depression, or epileptiform activity [3] (https://www.charite-academy.de/). Those who appreciate these subtleties may also understand that SD is much more than a simple change in brain activity and clearly more pathological to neurons than, for example, an epileptic seizure [4].

An important feature of SD is that it causes tone alterations in cerebral resistance vessels. For many decades, SD was believed to elicit only a single stereotyped hemodynamic response consisting of a brief, mild, and quite variable vasoconstriction, followed initially by marked vasodilation for approximately a minute and finally by mild vasoconstriction again, which then lasts for approximately an hour [5]. Importantly, brief SD that passes through metabolically intact tissues and produces this normal hemodynamic response does not cause irreversible neuronal damage, so SD, like an epileptic seizure, for example, can also be quite benign [6]. It was not until 1998 that it was discovered in animals that SD can also lead to a fundamentally different hemodynamic response in which severe vasoconstriction rather than vasodilation prevails during the phase in which neurons are depolarized and swollen, impeding neuronal recovery, prolonging both depolarization and cell swelling, and thus increasing the risk of neuronal death [7]. Through this mechanism of inverse hemodynamic response, SD can trigger spreading ischemia in previously nonischemic or mildly ischemic tissue, thereby inducing cerebral infarction [8]. Because subarachnoid erythrocytolysis produced this inversion of the hemodynamic response to SD, the authors hypothesized that this could be the mechanism of ischemic infarcts after SAH [7]. Importantly, among all currently known forms of cerebral vasospasm, this neuronally induced and neurovascular-unit-mediated form is the most severe [3]. The SD-induced vasospasm occurs acutely, spreads in the tissue, involves the entire microcirculation, and extends proximally at least to the pial arteries. The resulting spreading ischemia may last from less than a minute to several hours and is often followed by marked prolonged hyperemia, which may then revert to oligemia.

A controversy indirectly related to SAH that paralleled the above developments was the controversy between the so-called vascular hypothesis and the neuronal hypothesis of migraine aura. Wolff’s vascular hypothesis stated that migraine aura results from intracranial vasoconstriction, and Leão’s neuronal hypothesis postulated that SD-induced spreading depression of activity is the pathophysiologic correlate of migraine aura. Because SD is a primarily neuronal process, the two hypotheses were considered incompatible by the leading neurologists of their time. The majority actually assumed that SD does not occur in the human brain and is a subject of “neuromythology” [9]. However, the traditional controversy between the vascular and neuronal theories was scientifically settled in 2002 when it was discovered that brain topical administration of the vasoconstrictor polypeptide endothelin 1 is a highly effective trigger of SD in rodents in vivo and that endothelin 1 has this effect because of its vasoconstrictor properties, which cause SD in a concentration-dependent manner mediated by an imbalance between energy supply and demand of neurons [10]. Regarding SAH, this finding simultaneously implied that SDs arising in the setting of SAH could result not only from a direct action of blood on cortical cells but also indirectly as a consequence of vasospasm. In other words, as in migraine, vasospasm and SD are not mutually exclusive pathomechanisms in SAH but are complementary. In principle, vasospasm can cause SD and SD can cause vasospasm.

Also in 2002, Strong and colleagues [11] then introduced the first robust bedside method that, with subdural electrodes, detected SDs in approximately 50% of individuals with traumatic brain injury. In this way it was proven that SD is not a subject of neuromythology, and once again it was also shown that Charles Darwin was smarter than the average biped because extrapolation from one species to another works amazingly well when apples are compared to apples and not apples to oranges. The Co-Operative Studies on Brain Injury Depolarizations were then founded in 2003 (www.cosbid.org). In 2006, the first clinical study to demonstrate occurrence of SDs after SAH provided preliminary evidence that delayed ischemic neurological deficits after SAH are associated with a cluster of SDs [12]. In 2009, SD-induced spreading ischemia was detected in patients for the first time by using novel subdural optoelectrode technology for simultaneous laser Doppler flowmetry and direct current electrocorticography in combination with measurements of tissue partial pressure of oxygen after SAH [13]. In 2017, Hartings and colleagues [14] showed in a translational study that focal accumulation of subarachnoid blood is a sufficient insult to trigger SD clusters and early infarcts in a swine model and that, phenomenologically, nearly identical early neuromonitoring and neuroimaging findings occur in patients. In 2018, by using sophisticated neuromonitoring technology in combination with longitudinal neuroimaging, the entire sequence of both early and delayed brain infarct development after SAH with SD-induced persistent activity depression, SD-induced spreading ischemia, and the transition of clustered SDs to the negative ultraslow potential was demonstrated in a small patient population in which optoelectrodes were directly overlying newly developing infarcts [15] (https://www.youtube.com/watch?v=l06FWV9sowY).

In the same year, Sugimoto and colleagues [16] published a first-of-its-kind 50-patient treatment study of spreading ischemia with cilostazol, which stimulates nitrogen oxide production through endothelial NOS activation via a cAMP/PKA- and PI3K/Akt-dependent mechanism. This study showed a trend for less delayed cerebral ischemia (DCI) in the cilostazol group. Correspondingly, the total SD-induced depression duration per recording day and the occurrence of isoelectric SDs were significantly lower in the cilostazol group. In a companion study in rats published in the same article, cilostazol significantly shortened SD-induced spreading ischemia compared with vehicle. In this issue, Kawano, Sugimoto, and colleagues [17] now used data from this single-center randomized trial to explore the relationships of DCI with vasospasm, SD, and microcirculatory disturbance. Cerebral circulation time (CCT), which was divided into proximal CCT and peripheral CCT (as a measure of microcirculatory disturbance), was obtained from digital subtraction angiography (DSA) on day 9 ± 2 from onset. In univariate analysis, the number of SDs per day, the number of SDs on the day of DSA, and peripheral CCT were significant predictors of DCI, whereas the degree of angiographic vasospasm was not. Only the number of SDs on the day of DSA remained significant in multivariate analysis.

These findings add to the growing evidence that SDs play a central role in the pathogenesis of ischemic infarction after SAH and that SD variables may be a valuable predictor for treatment stratification during neurocritical care, particularly in patients who are comatose. However, larger multicenter studies are needed to explore this further.

In conclusion, contrary to the often expressed idea that SD is a homogeneous and stereotyped phenomenon, SD is one of the most complex and heterogeneous phenomena of the central nervous system, and because its nature has already been misjudged by generations of physicians and scientists, it continues to lead us by the nose. Slowly, we are coming to understand this. However, to truly change this for the benefit of our patients, further insight into the pitfalls of SD at all levels is needed by using all the technologies at our disposal, organotypic brain slice cultures, brain slices, animals with lissencephalic and gyrencephalic brains, theoretical models, machine learning approaches, and clinical investigations.
